# ^177^Lu-Prostate-Specific Membrane Antigen Therapy in Patients with Metastatic Castration-Resistant Prostate Cancer and Prior ^223^Ra (RALU Study)

**DOI:** 10.2967/jnumed.123.266125

**Published:** 2023-12

**Authors:** Kambiz Rahbar, Markus Essler, Matthias Eiber, Christian la Fougère, Vikas Prasad, Wolfgang P. Fendler, Philipp Rassek, Ergela Hasa, Helmut Dittmann, Ralph A. Bundschuh, Kim M. Pabst, Milena Kurtinecz, Anja Schmall, Frank Verholen, Oliver Sartor

**Affiliations:** 1 Department of Nuclear Medicine, University of Münster Medical Center, Münster, Germany;; 2 Department of Nuclear Medicine, University Hospital Bonn, Bonn, Germany;; 3 Department of Nuclear Medicine, Technical University of Munich, Munich, Germany;; 4 Department of Nuclear Medicine and Clinical Molecular Imaging, University Hospital Tübingen, Tübingen, Germany;; 5 Department of Nuclear Medicine, University of Ulm, Ulm, Germany;; 6 Department of Nuclear Medicine, German Cancer Consortium University Hospital Essen, Essen, Germany;; 7 Bayer HealthCare Pharmaceuticals, Whippany, New Jersey;; 8 Bayer Consumer Care, Basel, Switzerland; and; 9 Tulane Cancer Center, Tulane Medical School, New Orleans, Louisiana

**Keywords:** targeted α-therapy, ^223^Ra, ^177^Lu-PSMA, metastatic castration-resistant prostate cancer, real-world practice

## Abstract

^223^Ra-dichloride (^223^Ra) and ^177^Lu-prostate-specific membrane antigen (PSMA) are approved treatments for metastatic castration-resistant prostate cancer (mCRPC). The safety and effectiveness of sequential use of ^223^Ra and ^177^Lu-PSMA in patients with mCRPC are not well described. This study aimed to evaluate ^177^Lu-PSMA safety and efficacy in patients with mCRPC previously treated with ^223^Ra. **Methods:** The radium→lutetium (RALU) study was a multicenter, retrospective, medical chart review. Participants had received at least 1 ^223^Ra dose and, in any subsequent therapy line, at least 1 ^177^Lu-PSMA dose. Primary endpoints included the incidence of adverse events (AEs), serious AEs, grade 3–4 hematologic AEs, and abnormal laboratory values. Secondary endpoints included overall survival, time to next treatment/death, and change from baseline in serum prostate-specific antigen and alkaline phosphatase levels. **Results:** Data were from 133 patients. Before ^177^Lu-PSMA therapy, 56% (75/133) of patients received at least 4 life-prolonging therapies; all patients received ^223^Ra (73% received 5–6 injections). Overall, 27% (36/133) of patients received at least 5 ^177^Lu-PSMA infusions. Any-grade treatment-emergent AEs were reported in 79% (105/133) of patients and serious AEs in 30% (40/133). The most frequent grade 3–4 laboratory abnormalities were anemia (30%, 40/133) and thrombocytopenia (13%, 17/133). Median overall survival was 13.2 mo (95% CI, 10.5–15.6 mo) from the start of ^177^Lu-PSMA. **Conclusion:** In this real-world setting, ^223^Ra followed by ^177^Lu-PSMA therapy in heavily pretreated patients with mCRPC was clinically feasible, with no indication of impairment of ^177^Lu-PSMA safety or effectiveness.

## Visual Abstract

**Figure:**
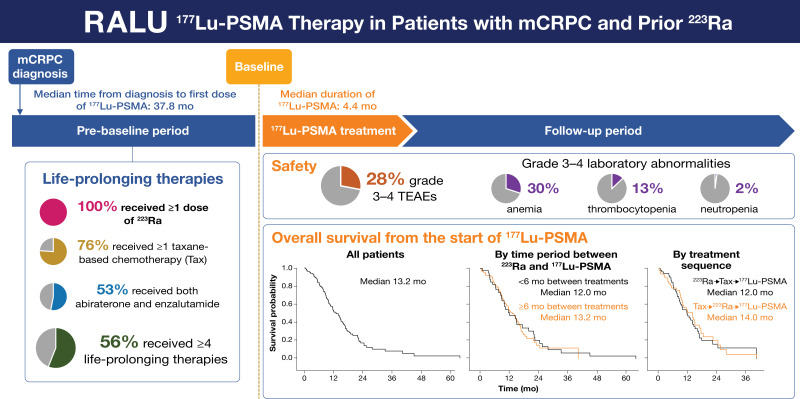

Metastatic prostate cancer is largely a disease of the bone (1), with a 5-y survival rate of less than 30% (2). For patients with metastatic castration-resistant prostate cancer (mCRPC), available life-prolonging therapies include taxane-based chemotherapy (docetaxel and cabazitaxel), androgen receptor pathway inhibitors (abiraterone and enzalutamide), the radionuclide ^223^Ra-dichloride (^223^Ra), the immunologic agent sipuleucel-T, the poly (adenosine diphosphate ribose) polymerase inhibitor olaparib, and ^177^Lu-vipivotide tetraxetan, consisting of a radionuclide (^177^Lu) linked to a ligand that binds to prostate-specific membrane antigen (PSMA), referred to as ^177^Lu-PSMA-617 hereafter (3–8).

^223^Ra is an α-particle–emitting radionuclide that mimics calcium and is incorporated into newly forming bone surrounding metastatic lesions (9*,*^10^). α-particles have high linear energy transfer, and the energy emitted by ^223^Ra decay causes DNA double-strand breaks that are difficult to repair (11). ^223^Ra is approved for the treatment of mCRPC with bone metastases but without visceral involvement (12*,*^13^). It is an established therapy that prolongs overall survival (OS) and has a favorable safety profile in this setting, as demonstrated in the phase 3 ALSYMPCA study (14*,*^15^). In contrast, ^177^Lu-PSMA-617 is a β-particle emitter that targets PSMA-expressing cells. On the basis of results from the phase 3 VISION study (16), ^177^Lu-PSMA-617 was recently approved in the United States and Europe for the treatment of PSMA-positive mCRPC in patients who have previously received docetaxel (7*,*^8^). In this trial, ^177^Lu-PSMA-617 therapy showed an acceptable safety profile and prolonged OS in patients heavily pretreated with other life-prolonging agents (in addition to docetaxel), including ^223^Ra (16).

Because of their differing mechanisms of action, there is considerable interest in understanding how ^223^Ra and ^177^Lu-PSMA-617 therapy at different time points in the therapeutic sequence may affect safety and efficacy outcomes. Notably, in VISION, patients (17.4%) treated with ^177^Lu-PSMA-617 who had previously received ^223^Ra therapy had benefits consistent with those of the overall patient population (17), although the safety for these patients has not yet been reported. However, small observational studies using ^177^Lu conjugated to various PSMA ligands indicate favorable toxicity and OS benefit in patients previously treated with ^223^Ra (18–20), including when ^177^Lu-PSMA therapy was initiated within 8 wk of stopping ^223^Ra (21).

Additional studies are required to provide further evidence of the safety and efficacy of sequential ^223^Ra and ^177^Lu-PSMA therapy. Here, we report findings from the radium→lutetium (RALU) study, which was designed to investigate the safety and efficacy of real-world ^177^Lu-PSMA use in patients with mCRPC who had previously received ^223^Ra. Additional post hoc analyses investigated whether ^177^Lu-PSMA safety or efficacy is impacted by the time between the last ^223^Ra dose and the first ^177^Lu-PSMA dose or prior use of both taxanes and ^223^Ra.

## MATERIALS AND METHODS

### Study Design

RALU is a multicenter, retrospective medical chart review study in Germany. This analysis reports final data from participating university hospitals. The study was conducted in accordance with guidelines and regulations of the European Medicines Agency and applicable local laws and regulations. The study protocol was approved by the institutional review board at each center. The requirement to obtain informed consent was waived because of the retrospective nature of the study and the fact that the patients were deceased or in a terminal disease stage.

### Patients

Patients whose medical records were evaluated in this study were not assigned to a particular therapeutic strategy. All treatment decisions documented in the patients’ medical records were made solely by the treating physicians in consultation with their patients as part of routine clinical practice. All treatment decisions were made within current clinical practice. Patients were assigned unique central identification codes, and only the treating physician, authorized site personnel, and authorized monitors and auditors had access to uncoded data.

Eligible patients were men aged at least 18 y, with a confirmed diagnosis of mCRPC, who had received at least 1 ^223^Ra dose and, in any subsequent therapy line, at least 1 ^177^Lu-PSMA dose. Chemotherapy before ^177^Lu-PSMA was allowed but not required. Exclusion criteria included previous treatment with hemibody radiation and radionuclide therapies other than ^223^Ra and ^177^Lu-PSMA. Patients were selected and their data collected by trained study-site personnel.

### Procedures

**FIGURE 1.**
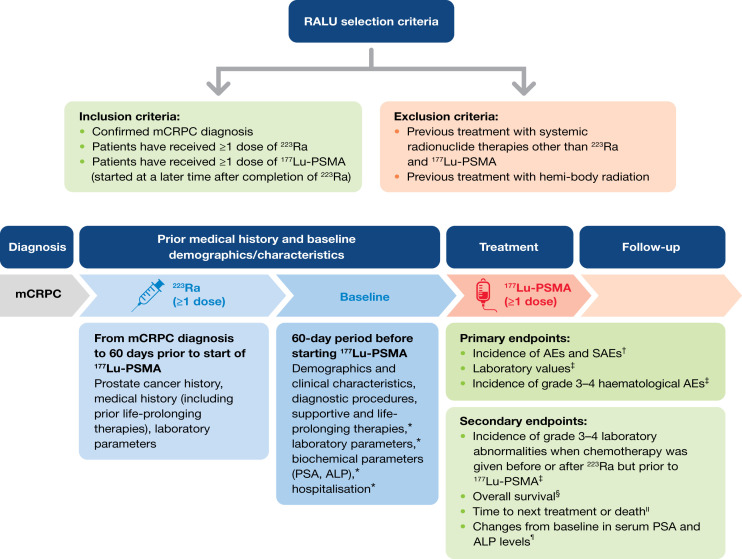
RALU study design. *Data also collected during ^177^Lu-PSMA therapy and during follow-up period after last ^177^Lu-PSMA dose. ^†^Measured from start of ^177^Lu-PSMA therapy up to 30 d after last dose. ^‡^Measured from start of ^177^Lu-PSMA therapy up to 90 d after last dose. ^§^Measured from both start of ^223^Ra and start of ^177^Lu-PSMA. ^‖^Measured from start of ^177^Lu-PSMA. ^¶^Measured during 60-d baseline period and during follow-up. SAE = serious AE.

Medical records were retrospectively reviewed between September 2021 and March 2022 (patients were treated between December 2014 and July 2021). The retrospective observation period started at mCRPC diagnosis and ended at the last available visit or death, whichever occurred first. The study design is shown in Figure 1. The radiation dose, activity, and date of each ^223^Ra injection were collected. ^177^Lu-PSMA therapy with any PSMA-targeted moiety was allowed, and the name or composition of the medicinal product, specification of the PSMA-targeted ligand, and radiation dose or activity and dates for each administration were collected. For chemotherapy exposure (docetaxel and cabazitaxel), the name and composition of the medicinal product, number of therapy cycles, calendar date for first and last administrations, and presence or absence of adverse events (AEs) were recorded. Use of other life-prolonging therapies was also documented (abiraterone acetate, enzalutamide, docetaxel, cabazitaxel, and ^223^Ra).

### Outcomes

The primary objective was to describe the safety of ^177^Lu-PSMA therapy in patients previously treated with ^223^Ra. Primary endpoints included the incidence of AEs and serious AEs, measured from the start of ^177^Lu-PSMA therapy up to 30 d after the last dose, and abnormal laboratory values (graded according to the Common Terminology Criteria for Adverse Events [version 5.0; AEs were graded retrospectively]) and grade 3–4 hematologic toxicities, measured from the start of ^177^Lu-PSMA therapy up to 90 d after the last dose. Secondary endpoints were the incidence of grade 3–4 laboratory abnormalities during ^177^Lu-PSMA treatment when chemotherapy was given before or after ^223^Ra therapy, OS from the start of ^177^Lu-PSMA treatment, time to next treatment or death, and changes from baseline in serum prostate-specific antigen (PSA) and alkaline phosphatase (ALP) levels measured during ^177^Lu-PSMA therapy.

Additional analyses were conducted for patients who received ^177^Lu-PSMA therapy for less than 6 mo versus 6 mo or more after the last ^223^Ra dose and for patients who received prior taxane-based chemotherapy in one of the following sequences: taxane-based chemotherapy, then ^223^Ra, then ^177^Lu-PSMA (Tax→Ra→Lu) or ^223^Ra, then taxane-based chemotherapy (during or after ^223^Ra), then ^177^Lu-PSMA (Ra→Tax→Lu).

### Statistical Analysis

Statistical analyses were exploratory; descriptive statistics were used. The study did not aim to confirm or reject predefined hypotheses. Summary statistics for duration of exposure and total number of treatment cycles were generated separately for ^177^Lu-PSMA, ^223^Ra, and chemotherapy. For OS and time to next treatment or death, median and 95% CIs were estimated using the Kaplan–Meier method. Summary statistics were generated for changes from baseline in ALP and PSA. No data imputation was planned.

## RESULTS

### Patients

In total, 133 patients with mCRPC were treated with ^223^Ra and subsequent ^177^Lu-PSMA. The patient disposition is shown in Supplemental Table 1 (supplemental materials are available at http://jnm.snmjournals.org). Baseline demographics and clinical characteristics at the start of ^177^Lu-PSMA therapy are provided in Table 1. Patients had an Eastern Cooperative Oncology Group performance status of either 1 (82/133, 62%) or 2 (51/133, 38%). Visceral metastases were present in 36 of 133 (27%) patients, with half of those having liver involvement (18/36, 50%). Median PSA and ALP values were 286 ng/mL and 146 U/L (Table 1).

**TABLE 1.** Baseline Demographics and Clinical Characteristics Before or at Start of ^177^Lu-PSMA

Characteristic	Data
Patients	133
Age (y)	73 (49–90)
ECOG PS	
1	82 (62)
2	51 (38)
PSA* (ng/mL) (*n* = 130)	286 (1–12,229)
ALP* (U/L) (*n* = 112)	146 (23–973)
Extent of metastatic disease^†^	
Bone metastases with lymph node metastases	63 (47)
Bone metastases without lymph node metastases	33 (25)
Visceral metastases	36 (27)
Prior therapies for mCRPC	
≥4 life-prolonging therapies^‡^	75 (56)
^223^Ra	133 (100)
Injections	
1–4	35 (26)
5–6	98 (74)
Abiraterone	95 (71)
Enzalutamide	92 (69)
Abiraterone and enzalutamide	71 (53)
Chemotherapy lines^§^	
0	31 (23)
1	67 (50)
≥2	35 (26)
Docetaxel	99 (74)
Cycles^‖^	
1–4	27 (24)
≥5	59 (53)
Missing/unknown	26 (23)
Cabazitaxel	30 (23)
Cycles^¶^	
1–4	7 (21)
≥5	14 (42)
Missing/unknown	12 (36)

* In case of multiple measures, value nearest to ^177^Lu-PSMA start was chosen.

† Prebaseline period, patient may have multiple metastatic diseases.

‡ Docetaxel, cabazitaxel, abiraterone, enzalutamide, ^223^Ra.

§ Chemotherapies with same start date ± 15 d were counted as 1 line.

‖ *n* = 112; percentages were based on number of docetaxel therapies (i.e., patients who received 2 lines of docetaxel were counted twice).

¶ *n* = 33; percentages were based on number of cabazitaxel therapies (i.e., patients who received 2 lines of cabazitaxel were counted twice).

ECOG PS = Eastern Cooperative Oncology Group performance status.

Qualitative data are number and percentage; continuous data are median and range.

Overall, 75 of 133 (56%) patients had received at least 4 life-prolonging therapies before starting ^177^Lu-PSMA (Table 1). All patients had received prior ^223^Ra; 98 (73%) had completed 5–6 injections (Table 1). Abiraterone and enzalutamide had been used to treat 95 (71%) and 92 (69%) patients, respectively; 71 (53%) patients had received both. In total, 102 (77%) patients had received at least 1 taxane-based chemotherapy (Table 1). The precise treatment sequence is known for 114 patients; ^223^Ra was the first therapy received during mCRPC for 23 of these 114 (20%) patients, second for 45 (40%), and third for 24 (21%) (Supplemental Fig. 1).

The median time from mCRPC diagnosis to the first ^177^Lu-PSMA dose was 37.8 mo (range, 7.4–161.5 mo), the median time from the last ^223^Ra injection to the first ^177^Lu-PSMA dose was 12.0 mo (range, 0.7–74.3 mo), and the median duration of ^177^Lu-PSMA therapy was 4.4 mo (range, 0.0–57.1 mo) (Table 2). Overall, 97 of 133 (73%) patients received 1–4 ^177^Lu-PSMA infusions and 36 (27%) received at least 5 infusions (Table 2); the median number of infusions was 4. After ^177^Lu-PSMA therapy, 76 (57%) patients died without receiving further treatment and 20 (15%) subsequently received further life-prolonging therapy (most commonly enzalutamide and abiraterone; no patients received ^223^Ra or ^177^Lu-PSMA). Among the subsequent treatment recipients, the median time to the next treatment was 1.1 mo (range, 0.0–2.8 mo). The median time to the next therapy or death from the start of ^177^Lu-PSMA therapy was 10.9 mo (95% CI, 9.1–13.2 mo), with 15 (11%) patients progressing to the next therapy or death within 30 d of the last ^177^Lu-PSMA dose.

**TABLE 2.** ^177^Lu-PSMA Therapy

Parameter	Data
Patients	133
Number of ^177^Lu-PSMA infusions	
1	17 (13)
2	33 (25)
3	16 (12)
4	31 (23)
5	13 (10)
6	14 (11)
>6	9 (7)
Duration of ^177^Lu-PSMA therapy (mo)	4.4 (0.0–57.1)
Time from last ^223^Ra injection to first ^177^Lu-PSMA dose (mo)*	12.0 (0.7–74.3)
Time from mCRPC diagnosis to first ^177^Lu-PSMA dose (mo)	37.8 (7.4–161.5)
Treatment delay/interruption/cessation^†^	51 (38)
Due to disease progression	23 (17)
Due to AE	13 (10)

* Last injection from first ^223^Ra treatment cycle in case of multiple cycles.

† Patients could have >1 delay and >1 reason for different delays.

Qualitative data are number and percentage; continuous data are median and range. Patients switched to another carrier molecule are reported under first carrier molecule.

### Safety

During ^177^Lu-PSMA treatment and up to 30 d after the last dose, any-grade treatment-emergent AEs (TEAEs) occurred in 105 of 133 (79%) patients and serious TEAEs in 40 (30%); grade 3–4 and grade 5 TEAEs occurred in 37 (28%) and 5 (4%) patients, respectively (Table 3). In patients who received ^177^Lu-PSMA within 6 mo versus 6 mo or more after completing ^223^Ra, any-grade TEAEs were reported in 30 of 42 (71%) versus 74 of 90 (82%) patients, respectively; corresponding values for serious TEAEs were 14 of 42 (33%) versus 25 of 90 (28%) (Table 3). Overall, anemia was the most common grade 3–4 TEAE (20/133, 15%; Supplemental Table 2) and serious TEAE (16/133, 12%; Supplemental Table 3); 2 of 133 (2%) patients had grade 3–4 thrombocytopenia (Supplemental Table 2). One patient (1%) experienced a pathologic fracture (grade 1–2).

**TABLE 3.** TEAEs During ^177^Lu-PSMA Therapy

		Time between end of ^223^Ra and start of ^177^Lu-PSMA*	
Event	All patients (*n* = 133)	<6 mo (*n* = 42)	≥6 mo (*n* = 90)
TEAE			
Any grade	105 (79%)	30 (71%)	74 (82%)
Grade 3–4	37 (28%)	15 (36%)	22 (24%)
Serious	40 (30%)	14 (33%)	25 (28%)
TEAEs in ≥10% of patients by PT^†^			
Dry mouth			
Any grade	20 (15%)	3 (7%)	16 (18%)
Grade 3–4	0 (0%)	0 (0%)	0 (0%)
Nausea			
Any grade	12 (9%)	5 (12%)	7 (8%)
Grade 3–4	0 (0%)	0 (0%)	0 (0%)
Fatigue			
Any grade	11 (8%)	5 (12%)	6 (7%)
Grade 3–4	0 (0%)	0 (0%)	0 (0%)

* Time between therapies was unknown in 1 patient and therefore not included in this analysis.

† Categories selected by incidence of TEAEs in any group. Excludes laboratory abnormalities.

CTCAE = Common Terminology Criteria for Adverse Events; MedDRA = Medical Dictionary for Regulatory Activities; PT = preferred term.

Data are from start of ^177^Lu-PSMA treatment to 30 d after last dose. MedDRA PT is by CTCAE grading.

The most common grade 3–4 laboratory abnormalities were anemia (40/133; 30%) and thrombocytopenia (17/133, 13%) (Table 4). Of the patients with grade 3–4 thrombocytopenia, 8 of 17 (47%) had preexisting thrombocytopenia before receiving ^177^Lu-PSMA treatment. The incidence of grade 3–4 laboratory abnormalities was similar irrespective of whether patients received ^177^Lu-PSMA less than 6 mo or 6 mo or more after their last ^223^Ra dose (Table 4). Notably, 25 of 42 (60%) patients received ^177^Lu-PSMA within 12 wk after ^223^Ra in the less-than-6-mo subgroup.

**TABLE 4.** Incidence of Grade 3–4 Laboratory Abnormalities During ^177^Lu-PSMA Therapy

Abnormality	All patients	Time between end of ^223^Ra and start of ^177^Lu-PSMA*	
		<6 mo	≥6 mo
Anemia	40/133 (30%)	14/42 (33%)	25/90 (28%)
Thrombocytopenia	17/133 (13%)^†^	5/42 (12%)	12/90 (13%)
Neutropenia	3/130 (2%)	2/41 (5%)	1/88 (1%)
ASAT elevation	2/131 (2%)	1/42 (2%)	1/88 (1%)

* Time between therapies was unknown in 1 patient and therefore not included in this analysis.

† Eight of 17 patients had thrombocytopenia at start of ^177^Lu-PSMA.

ASAT = aspartate aminotransferase.

Data are from start of ^177^Lu-PSMA treatment to 90 d after last dose.

### Efficacy

**FIGURE 2.**
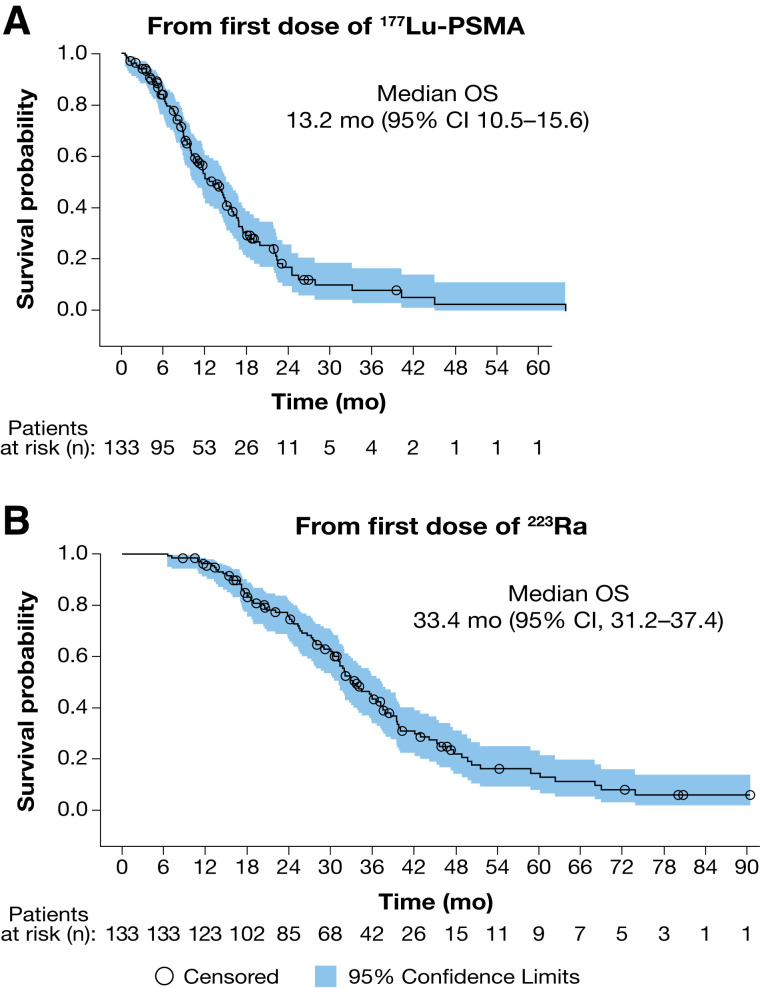
Kaplan–Meier estimates of OS from first dose of ^177^Lu-PSMA (A) and first dose of ^223^Ra (B). Descriptive statistics and Kaplan–Meier survival probabilities were generated.

**FIGURE 3.**
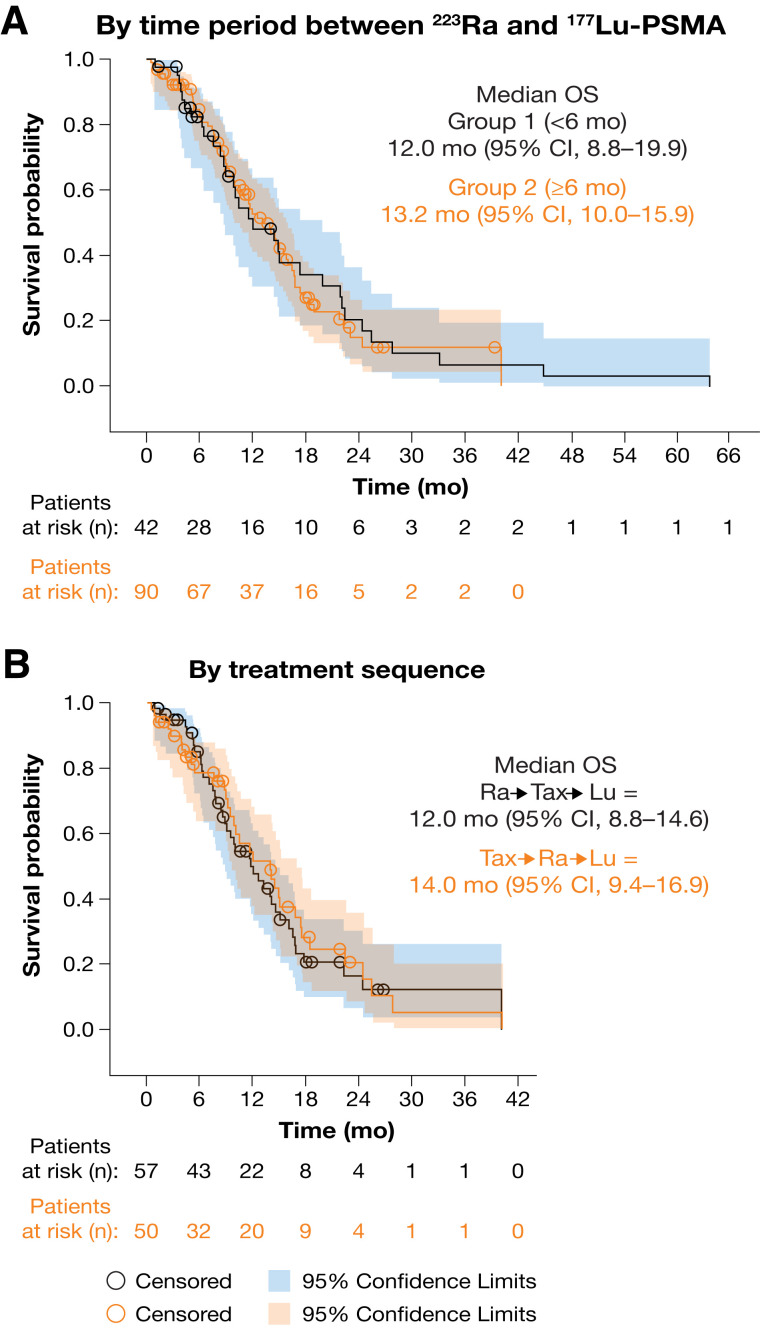
Kaplan–Meier estimates of OS by time between end of ^223^Ra and start of ^177^Lu-PSMA (A) and from first dose of ^177^Lu-PSMA by treatment sequence (B). Descriptive statistics and Kaplan–Meier survival probabilities were generated. OS was calculated from first dose of ^177^Lu-PSMA. For treatment sequence analysis, 13 patients with multiple taxane-based chemotherapy treatments administered before and after ^223^Ra were included in both groups; patients who did not receive taxane-based chemotherapy (31/133, 23%) were excluded.

Median OS calculated from the first ^177^Lu-PSMA dose was 13.2 mo (95% CI, 10.5–15.6 mo) (Fig. 2A); OS was similar irrespective of whether patients received ^177^Lu-PSMA less than 6 mo (12.0 mo [95% CI, 8.8–19.9 mo]) or 6 mo or more (13.2 mo [95% CI, 10.0–15.9 mo]) after their last ^223^Ra dose (Fig. 3A). When calculated from the first ^223^Ra dose, median OS was 33.4 mo (95% CI, 31.2–37.4 mo) (Fig. 2B).

Overall, 55 of 133 (41%) patients had available PSA data during ^177^Lu-PSMA treatment; a PSA decline of at least 30% or at least 50% occurred in 26 (47%) and 23 (42%) patients, respectively. Corresponding ALP declines occurred in 9 (19%) and 4 (9%) of 47 patients.

### Safety and Efficacy of ^177^Lu-PSMA When Both ^223^Ra and Chemotherapy Were Prior Therapies

Analyses were conducted on patients who had received both taxane-based chemotherapy and ^223^Ra before ^177^Lu-PSMA. Patients were grouped depending on whether they had received taxane-based chemotherapy during or after ^223^Ra treatment (Ra→Tax→Lu; *n* = 57) or before (Tax→Ra→Lu; *n* = 50). Baseline characteristics of these subgroups are reported in Supplemental Table 4. Of the 57 patients who received taxane-based chemotherapy during or after ^223^Ra treatment, 6 patients started chemotherapy during ^223^Ra therapy and 51 started chemotherapy after ^223^Ra therapy. Any-grade and grade 3–4 TEAEs occurred in 50 of 57 (88%) and 14 of 57 (25%) patients in the Ra→Tax→Lu group and in 39 of 50 (78%) and 17 of 50 (34%) patients in the Tax→Ra→Lu group, respectively (Supplemental Table 5). A lower proportion of patients had grade 3–4 laboratory abnormalities of anemia and thrombocytopenia in the Ra→Tax→Lu group (15/57 [26%] and 7/57 [12%], respectively) than in the Tax→Ra→Lu group (19/50 [38%] and 9/50 [18%], respectively; Supplemental Table 6). Median OS from the first ^177^Lu-PSMA dose was 12.0 mo (95% CI, 8.8–14.6 mo) in the Ra→Tax→Lu group and 14.0 mo (95% CI, 9.4–16.9 mo) in the Tax→Ra→Lu group (Fig. 3B).

## DISCUSSION

Here we show that ^177^Lu-PSMA therapy in patients with mCRPC previously treated with ^223^Ra is clinically feasible, with safety and survival outcomes similar to those reported in the VISION trial (16), other smaller studies (19–22), and a preplanned interim analysis of the current study (23).

Although ^223^Ra and ^177^Lu-PSMA were associated with a low incidence of myelosuppression in randomized trials (14*,*^16^*,*^24^), the potential risk of increased hematologic AEs requires consideration when using sequential systemic radionuclide therapies, particularly in the context of chemotherapy exposure and advanced disease (19*,*^25^*,*^26^). The current study provides important insights into the feasibility of sequencing radiopharmaceuticals. The incidence of Common Terminology Criteria for Adverse Events grade 3–4 anemia and thrombocytopenia (15% and 2%) in RALU was consistent with the results (15% and 4%) of the prospective noninterventional REASSURE study in patients who received ^177^Lu-PSMA subsequent to ^223^Ra (22) and the results (13% and 8%) of the ^177^Lu-PSMA-617 arm of the prospective interventional VISION trial (16). These findings suggest that using ^223^Ra and ^177^Lu-PSMA sequentially is not associated with cumulative hematologic toxicity.

Our results show that the safety profile and OS outcomes of ^177^Lu-PSMA were similar whether taxanes were used before or after ^223^Ra. Furthermore, 59% of patients received ^223^Ra early in the treatment sequence (first or second line after mCRPC diagnosis). Thus, receiving ^223^Ra earlier in the treatment sequence did not prevent these patients from receiving subsequent cytotoxic therapies such as taxanes or ^177^Lu-PSMA. Further studies on the optimal use of available therapies are warranted, including combined (27) or alternating use of ^223^Ra and ^177^Lu-PSMA (given their complementary mechanisms of action).

These data provide physicians with important information regarding the hematologic safety of receiving 2 systemic radiopharmaceuticals. Because physicians with different medical specialties or disciplines manage patients at this advanced stage, interaction and communication within a multidisciplinary medical team (including radiologists, nuclear medicine physicians, radiology nurses, oncologists, and urologists) is important to ensure that patients are receiving the best available care. This was conveyed in an expert recommendation paper from nuclear medicine centers across Europe, in which guidance on optimizing ^223^Ra use was delineated and outlined (28).

Our findings require consideration of the study limitations, among which are its retrospective design (potentially contributing to patient selection bias), lack of a control arm, and data that are from a single country. However, our study is strengthened by its relatively unconstrained inclusion criteria, the fact that there are few missing data, and the fact that patients were treated in nuclear medicine centers with extensive experience with ^223^Ra and ^177^Lu-PSMA.

## CONCLUSION

In this real-world setting of patients with mCRPC previously treated with ^223^Ra, ^177^Lu-PSMA therapy had an acceptable safety profile and effectiveness comparable to that seen in the VISION trial. These data also support the feasibility of giving ^177^Lu-PSMA within 6 mo of completing ^223^Ra therapy. Furthermore, safety and OS outcomes were similar regardless of the order of chemotherapy use in the sequence (e.g., before or after ^223^Ra). These findings may inform decision making when considering treatment strategies for patients with mCRPC and bone metastases, with the ultimate goal of prolonging life using the right treatment at the right time.

## DISCLOSURE

This work was supported by Bayer AG. Kambiz Rahbar reports honoraria from Advanced Accelerator Applications (AAA) and Bayer and a consultancy/advisory role with ABX GmbH, ABX-CRO, Bayer, and AAA. Markus Essler reports a consultancy/advisory role with Bayer, AAA, and Ipsen and travel expenses from Ipsen. Matthias Eiber reports stocks/other ownership interests in Novartis and Telix Pharmaceuticals; a consultancy/advisory role with Blue Earth Diagnostics, ABX Advanced Biochemical Compounds, Janssen Oncology, Telix Pharmaceuticals, and Novartis; research funding from Siemens, ABX Advanced Biochemical Compounds, Blue Earth Diagnostics, and Bayer; a patent application for rhPSMA; and travel expenses from Bayer Schering Pharma. Christian la Fougère reports a consultancy/advisory role with Bayer, Novartis, EUSA-Pharma, Ipsen, Oncodesign, and Sirtex Medical and research funding from Oncovision and Siemens Healthineers. Vikas Prasad reports honoraria from AAA, a consultancy/advisory role with Bayer, and research funding from Ipsen. Wolfgang Fendler reports honoraria from Parexel and AAA; a consultancy/advisory role with Janssen, Calyx, and Bayer; and research funding from SOFIE. Philipp Rassek is an employee of Porterhouse Group AG Paracelsus Kliniken. Helmut Dittmann reports a consultancy/advisory role with Bayer, Ipsen, and Eisai AG. Ralph Bundschuh reports honoraria from Eisai AG and a consultancy/advisory role with Bayer. Kim Pabst has received a Junior Clinician Scientist Stipend of the University Medicine Essen Clinician Scientist Academy (sponsor: Faculty of Medicine and Deutsche Forschungsgemeinschaft), research funding from Bayer, and travel fees from Ipsen. Milena Kurtinecz, Anja Schmall, and Frank Verholen are employees of Bayer. Oliver Sartor reports a consultancy/advisory role with Bayer, Sanofi, AstraZeneca, Dendreon, Constellation Pharmaceuticals, AAA, Pfizer, Bristol-Myers Squibb, Bavarian Nordic, EMD Serono, Astellas Pharma, Progenics, Blue Earth Diagnostics, Myovant Sciences, Myriad Genetics, Novartis, Clarity Pharmaceuticals, Fusion Pharmaceuticals, Isotopen Technologien, Janssen, Noxopharm, Clovis Oncology, Noria Therapeutics, Point Biopharma, TeneoBio, Telix Pharmaceuticals, and Theragnostics; travel expenses from Bayer, Johnson & Johnson, Sanofi, AstraZeneca, and Progenics; expert testimony for Sanofi; stocks/other ownership interests in Lilly, GlaxoSmithKline, Abbvie, Cardinal Health, United Health Group, PSMA Therapeutics, Clarity Pharmaceuticals, Noria Therapeutics, and Clovis Oncology; and research funding from Bayer, Sanofi, Endocyte, Merck, InVitae, Constellation Pharmaceuticals, AAA, AstraZeneca, Dendreon, SOTIO, Janssen, and Progenics. Chris Guise of Cancer Communications and Consultancy Ltd, Cheshire, U.K., provided medical writing assistance funded by Bayer. Lila Adnane (Bayer) provided editorial assistance. No other potential conflict of interest relevant to this article was reported.

## Notes

KEY POINTS **QUESTION:** Can ^177^Lu-PSMA be safely given to patients with mCRPC if they have previously received ^223^Ra, and is safety impacted depending on where ^223^Ra is positioned in the treatment sequence? **PERTINENT FINDINGS:** In this real-world setting, ^177^Lu-PSMA had an acceptable safety profile in patients who had previously received ^223^Ra, with low rates of hematologic and overall AEs. Median OS from the first dose of ^177^Lu-PSMA was 13.2 mo and was similar irrespective of whether patients had received taxane-based chemotherapy before or after ^223^Ra or if the time between ^223^Ra and ^177^Lu-PSMA was less than 6 mo versus 6 mo or more. **IMPLICATIONS FOR PATIENT CARE:** In patients with mCRPC and prior ^223^Ra therapy, ^177^Lu-PSMA had an acceptable safety profile and an effectiveness comparable to that seen in the VISION trial, irrespective of when patients had received prior ^223^Ra.
